# Host diets drive microbiome evolution and functional divergence

**DOI:** 10.1016/j.gendis.2026.102068

**Published:** 2026-02-04

**Authors:** Ka Lam Nguyen, Jason Sun Xia, Wenyan Mei

**Affiliations:** a Department of Comparative Biosciences, College of Veterinary Medicine, University of Illinois Urbana-Champaign, Urbana, IL 61802, USA; b Cancer Center at Illinois, University of Illinois Urbana-Champaign, Urbana, IL 61801, USA; c Carl R. Woese Institute for Genomic Biology, University of Illinois Urbana-Champaign, Urbana, IL 61801, USA; d Division of Nutritional Sciences, University of Illinois Urbana-Champaign, Urbana, IL 61801, USA

**Keywords:** Diet, Functional divergence, Genetic adaptation, Gut microbiome evolution, Health impact, Metabolic capability

## Abstract

Substantial variation in gut microbiome function across individuals underscores the pivotal role of the host in shaping microbial diversity. Increasing evidence indicates that diet is a major selective force driving the evolutionary trajectory of the gut microbiome. In response to dietary inputs, the microbiome adapts by shifting the relative abundance of taxa, species, and sub-strains. The gut microbiome also acquires novel genetic features through selecting advantageous mutations and genes, as well as colonizing exogenous bacterial species that enhance nutrient utilization. These adaptive processes occur not only across human evolutionary history but also within an individual's lifetime, consequently generating functionally divergent microbiomes with important consequences for health. Diets of poor nutritional quality, such as the Western-style diet, foster disease-permissive microbial functions. Understanding how dietary patterns and components influence microbiome evolution, and thereby affect health, is essential for developing diet-based strategies to modulate microbial functions. This review presents compelling evidence to demonstrate how host diet shapes gut microbiome evolution and leads to a functionally divergent gut microbiome that impacts human health.

## Introduction

The gut microbiota is the collection of microorganisms that live in the gut, comprising bacteria, archaea, viruses, and fungi. With a staggering collection of more than 20 million non-redundant microbial genes, the gut microbiota represents an additional metabolic organ that contributes substantially to human health.^1^ Several core functions of the gut microbiome include fermenting indigestible dietary fiber into various bioactive metabolites, synthesizing vitamins, generating colonization resistance to pathogens, and promoting intestinal epithelium homeostasis and immune system maturation.^2^, ^3^, ^4^, ^5^, ^6^, ^7^ Despite the core functions, the gut microbiome exhibits substantial variability across individuals based on age, genetics, cultural traditions, diet, and geographical location.^8^, ^9^, ^10^ This variation in the human gut microbiome has been linked to differential disease susceptibility and responses to therapy.^11^, ^12^, ^13^, ^14^, ^15^, ^16^, ^17^ As such, investigating factors that promote the functional divergence of the gut microbiome is vital for prevention, early diagnosis, and effective treatment of human diseases.

Accumulating evidence suggests that the host diet is one of the major factors that shape the gut microbiome evolution and cause microbiome functional divergence. The diet-induced selective pressure on gut microbiome evolution is conserved across the mammalian phylogeny.^18^^,^^19^ The complexity of species-specific carbohydrate diets is particularly important in shaping the metabolic repertoires of the mammalian gut microbiome.^19^^,^^20^ One of the genetic adaptations to specific carbohydrate diets is the acquisition of microbial genes encoding digestive enzymes specific to such carbohydrates. For instance, Burkina Faso children consuming a rural West African high-fiber diet have *Prevotella* spp. and *Xylanibacter* spp. in their gut microbiota. These species have acquired genes encoding xylanase and carboxymethylcellulase that ferment xylan and cellulose.^21^ In contrast, these species are absent in the age-matched gut microbiome of European children consuming a Western-style low-fiber diet.^21^ Host diets also influence the natural selection of genetic mutations in the gut microbiome. For instance, the types of genetic mutations accumulated in *Bacteroides thetaiotaomicron* (*B. theta*) in the mouse gut correlate with the amount of dietary fiber intake.^22^ A high-fiber diet selects mutations in *B. theta* that favor fiber degradation, whereas a low-fiber diet selects mutations that promote the degradation of host mucin-derived glycans.^22^ In this review, we highlight compelling evidence to show the influence of host diets on gut microbiome evolution and its impact on microbiome function and host health.

### Microbiome evolution and functional divergence

Gut microbiome evolution is the change in the genetic characteristics of the gut microbes to adapt to the changing environments over time. The host provides carbohydrates to gut microbes through consuming diets that contain microbiota-accessible carbohydrates (MACs). Most simple carbohydrates are absorbed by the host before reaching the colon.^23^ Thus, dietary fiber, a diverse group of carbohydrate polymers indigestible to the host, is an important source of MACs.^24^^,^^25^ Since host diets predominantly determine the MAC availability to gut microbes,^26^ adaptations to different host diets have become a major driving force for gut microbiome evolution.^27^, ^28^, ^29^, ^30^ These adaptive processes occur both across human evolutionary history and within an individual's lifespan,^30^, ^31^, ^32^, ^33^ and are reflected at the levels of microbial community structure and gene content. In this review, we highlight both types of changes, with a particular emphasis on gene-level mechanisms of adaptation.

### Structural adaptation of the gut microbiota

Genetic adaptation at the structural level is often reflected in the changes of gut microbiota composition, such as altered diversity and proportions of taxa, variants, and sub-strains within one taxon. Diet patterns drive these changes in the gut microbiota across both short and long timescales but have distinct modes of influence. Diet-induced alterations in microbiota structure also vary markedly among individuals, underscoring substantial inter-individual variability in microbial responsiveness. These concepts are supported by several elegant studies that have examined how gut microbial communities evolve under different dietary conditions and over varying durations.

### Long-term and short-term diet effects on gut microbiota

In a 6-year study using mice colonized with 12 representative taxa, Yilmaz et al have discovered that, under constant environmental and nutritional conditions, there is slow evolution through a progressive positive selection in individual taxa and microbial sub-strains.^30^ Interestingly, evolved sub-strains within a single taxon coexist in a stable equilibrium. Diet shift causes a rapid change of sub-strain proportions within a taxon by selecting sub-strain variants that allow a quick adaptation to new dietary conditions. For example, when mice were transitioned from chow to a high-fat diet, microbial gene variants encoding enzymes involved in carbohydrate metabolism exhibited pronounced frequency changes, likely indicating genetic adaptation to the altered carbon source.^30^

In another study, Sonnenburg et al investigated the short-term and long-term effects of a low-MAC diet on gut microbiota composition by tracking humanized mice (mice colonized with human microbiota) across four generations.^34^ They show that a low-MAC diet reduces the gut microbiota diversity. This reduction is largely reversible within a single generation following the reintroduction of dietary MACs. However, persistent low-MAC diets across generations cause a progressive and irreversible loss of microbial diversity, leading to a reduced diversity of glycoside hydrolases required for glycan degradation. This important finding reveals a deleterious effect of a long-term low-MAC diet on the carbohydrate metabolism capacity of gut microbiota.^34^

A human trial conducted by Li et al supports the idea that short-term dietary effects on the gut microbiota are transient and reversible.^35^ They evaluated whether a three-week non-industrialized dietary intervention, characterized by a plant-based and high-fiber diet, could restore gut microbiome features altered by industrialization in healthy Canadian adults. The intervention resulted in significant shifts in microbial composition, including an increase in putative health-promoting species and a decrease in putative pro-inflammatory species. The ratio of microbe-encoded carbohydrate-active enzymes targeting plant-derived carbohydrates relative to those involved in mucus degradation is markedly increased. Further, the dietary intervention improved both fecal and plasma metabolomic profiles and reduced risk markers associated with non-communicable diseases such as obesity, type 2 diabetes, and cardiovascular diseases. However, upon returning to their usual diet, participants' gut microbiota rapidly reverted to baseline states, suggesting that the beneficial effects of such short-duration dietary intervention were not sustained.

Together, these studies highlight the dynamic yet resilient nature of the gut microbiota in response to dietary inputs. Short-term dietary interventions can transiently modulate microbial composition and function, leading to temporary health benefits. However, sustained dietary patterns exert a far greater influence on gut microbiota function. As such, maintaining long-term, nutrient-rich dietary habits may be essential not only for promoting a diverse and metabolically capable microbiota but also for ensuring the persistence of health-associated microbial traits across time and generations.

### Inter-individual variation in diet-induced gut microbiota compositional change

One unexpected finding from the studies of Li et al and others is that the dietary effect on gut microbiota composition is highly individualized, and a dietary pattern alone has a limited impact on reducing gut microbiota inter-individual variation, especially in functional features such as metabolic modules.^35^, ^36^, ^37^, ^38^, ^39^ For example, Huang et al investigated the strain-level difference in gut microbiota composition across individuals following vegan, flexitarian, and omnivorous diets.^38^ They show that while the dietary pattern significantly influences individual species abundance and strain genetic variation, it is insufficient to differentiate inter-individual variations in the whole microbiome functional features. By comparing their results with those from other studies, they identified only a limited subset of species and strain-level genetic changes that consistently respond to the three dietary patterns. This includes a reduction in the genetic variation of vitamin B12-dependent ribonucleotide reductase in vegans and an increase in the genetic variation of genes involved in iron transport in omnivores. However, no consensus changes were found in genes encoding carbohydrate-active enzymes. A similar finding was reported by Shetty et al who investigated the gut microbiome of Dutch individuals following vegan, vegetarian, pescatarian, and omnivorous diets.^39^ Such an individualized dietary effect on gut microbiome may be attributed to functional redundancy among unrelated gut microbiota members.^38^, ^39^, ^40^ Additionally, the presence of common dietary components, along with other confounding factors such as age, genetics, and lifestyle, may obscure the specific effects of diet on microbial evolution.

Collectively, these human and animal studies indicate that diet shapes the structural and evolutionary trajectory of the gut microbiota by modulating the abundance and composition of taxa, species, and sub-strains. These effects, however, are highly individualized on the gut microbiota composition (Table 1).

**Table 1:** Summary of key findings on diet-induced gut microbiota change.

Dietary patterns	Key feature(s)	Genetic changes	Evolutionary insights
Vegan diet 36, 37, 38, 39	Plant-based; high dietary fiber; no vitamin B12	• Enriched fiber-consuming species • Decreased genetic variations of vitamin B12- dependent ribonucleotide reductase • Diet-associated species changes are highly individualized	• Availability of specific dietary components increases genetic variations and enhances the metabolizing capability of gut microbiota on these substrates. • Absence of specific dietary components causes loss of the gut microbiota's capability of metabolizing these substrates. • Diet pattern alone is insufficient to reduce gut microbiota inter-individual variation, and other factors may contribute to gut microbiota responsiveness to diet.
Omnivorous diet 36, 37, 38, 39	Diverse, flexible nutrient intake; greater access to iron and vitamin B12	• Enriched animal-derived food-consuming species • Increased genetic variations of genes essential for iron transport • Diet-associated species changes are highly individualized	
Coastal diet^42^^,^^43^^,^^122^	Seaweed-based; rich in marine polysaccharides	• The gut microbiome has acquired genes encoding enzymes, regulators, and transporters for degrading seaweed polysaccharides by HGT or exogenous bacteria engraftment.	• Introducing new dietary components increases microbiome genetic variations • Multiple adaptive mechanisms contribute to assembling new microbial functional guilds capable of utilizing the novel dietary component.
Western-style diet ^22^^,^^30^^,^^34^^,^^41^^,^^47^^,^^48^^,^^50^^,^^74^, ^75^, ^76^, ^77^, ^78^	Low dietary fiber, high fat, high taurine	• Structurally, a persistent low-MAC diet causes irreversible loss of microbial diversity and a reduction in carbohydrate metabolism capacity. • At the gene level, a low-MAC diet selects mutations or acquires genes by HGT that enhance mucin utilization. • A high-fat and high-taurine diet alters the BA pool and, consequently, may select gut microbes that are resistant to bile acids and can deconjugate taurine.	• Low-MAC diets decrease the capability of fiber metabolism and enhance mucin utilization. • High fat and high taurine enhance the bile acid deconjugation capacity of gut microbiota and resistance to bile acids.
Fluctuating diets^30^^,^^34^	Long-term chow diet shifts to starch or high-fat diets.	• Constant nutrition conditions drive progressive positive selection in individual taxa and microbial sub-strains.s • Evolved sub-strains within a single taxon coexist in a stable equilibrium. • Diet shift selects sub-strain variants for the adaptation to new dietary conditions.	• The coexistence of evolved microbial sub-strains enhances genomic flexibility, thereby promoting improved adaptation to novel dietary conditions.

### Gene-level adaptation of the gut microbiota

Genetic adaptations at the gene level in response to diet involve a range of mechanisms, including mutation selection and gene acquisition via horizontal gene transfer (HGT).^22^^,^^41^, ^42^, ^43^ These processes enable microbes to rapidly adjust their metabolic capacities to meet changing nutritional pressures. In the following sections, we highlight key examples of mutation selection and HGT to address two central questions: how do specific dietary components drive gut microbiome evolution, and how do these resulting genetic adaptations influence human health.

### The impact of low MACs

MACs in dietary fiber serve as essential nutrient sources not only for fiber-fermenting gut microbes but also for microbes that rely on metabolites released through fiber fermentation, such as via cross-feeding interactions. As such, the availability of dietary MAC imposes strong selective pressure on gut microbiome evolution, particularly on genes involved in carbohydrate metabolism, leading to the selection of advantageous mutations or the acquisition of novel genes (Table 2).

**Table 2:** Summary of key findings on diet-induced adaptive mutations.

Fitness advantage	Diet and affected genes	Bacterial strain and model system used	
Loss of function mutations in *ara*C, *malI*, and *melR* increased colonization fitness, especially under a high-MAC diet^41^	• Loss-of-function mutations enriched exclusively under the high-MAC diet: *malI*, *melR*, *ygjI* • Nonsynonymous or loss-of-function mutations enriched exclusively under the low-MAC diet: *ssuC*, *nupG*, *cueO*, *elaD*, *gntR*, *yidE*, and *envZ* • Nonsynonymous or loss-of-function mutations enriched under both the high-MAC and Low-MAC diets: *gatC*, *araC*, *dgsA,* and *ygfS*	Hyper-mutator strain of *E. coli* K-12, mono-colonized mice	
Enhances mucin utilization^47^	Loss-of-function mutations enriched under the high-fat diet: *nagC* and *gntT*, increasing the ability to use GlcNAc and reducing the ability to utilize gluconate	*E. coli* Nissle 1917, mono-colonized or 13-gut microbiota member colonized mice	
Increased colonization fitness under WD^22^	Missense mutations enriched under the Western-style diet: *susC-like* and *susD-like* that target mucin o-glycans	*B. theta* in specific-pathogen-free mice pretreated with antibiotics	
Increased colonization fitness under SD^22^	Mutations enriched under the chow diet: the hybrid two-component system that targets fructans		
Better evolutionary adaptation to a fluctuating dietary regimen^22^	Mutations and loss-of-function mutations enriched under alternative diets: *PUL-associated ORF* that target mucin o- glycans, *susD* that target starches, *susD-like* that target arabinan, *gh43*, and the *hybrid two-component system* that target arabinan, and pectin galactan		
Enhanced resistance to bile salts 82, 83, 84, 85	Missense mutations in *envZ* are enriched under BA-induced high osmotic conditions	*E. coli* strains, mono-colonized mice, or *in vitro* analysis	

### Mutation selection

Most evidence on mutation selection came from the studies using *B. theta* and *Escherichia coli* (*E. coli*) strains as model systems.

### Mutation selection in *B. theta*

*B. theta* is a member of fiber-degrading *Bacteroides* prevalent in the human gut. *B. theta* consumes host glycans if dietary plant polysaccharides are inaccessible.^22^^,^^44^ In an elegant study conducted by Dapa et al, they studied the evolutionary dynamics of *B. theta* over a 3-month timescale under different diets.^22^ They treated mice raised under specific pathogen-free conditions with antibiotics ciprofloxacin HCl and metronidazole to enable *B. theta* colonization. They fed *B. theta*-colonized mice a standard chow diet (SD) containing high MACs, a Western-style diet (WD) containing low MACs, or a diet alternating (AD) between SD and WD each week (Readers can refer to the cited paper for detailed dietary compositions of each diet).^22^ They found that *B. theta* from both SD- and WD-fed mice outcompeted their ancestral strain in colonizing mice. Further, the SD-evolved *B. theta* outcompeted the WD-evolved *B. theta* in mice fed with SD, and *vice versa*. On the contrary, *B. theta* in mice fed with AD was under a fluctuating selection correlating to the weekly diet change. This interesting finding indicates that *B. theta* with adaptive mutations to specific diets were selected because of their superior colonization fitness in the gut.

By comparing the genome of evolved *B. theta* to that of their ancestral strain, Dapa et al show that most mutations in evolved *B. theta* are missense or synonymous, likely affecting the levels and activities of the encoded proteins. Further, some mutations are diet-specific.^22^ For instance, the WD-evolved *B. theta* has enriched mutations in *(sus)C*-like and *(sus)D*-like genes that target dietary substrate mucin o-glycans, whereas the SD-evolved *B. theta* is enriched with mutations in gene that targets fructans. Interestingly, the AD-evolved *B. theta* contained more mutational targets than those of SD- or WD-evolved *B. theta* populations alone, including genes that target a variety of substrates such as mucin o-glycans, starches, arabinan, and pectin galactan. This leads to an increased intraspecies genetic diversity, which presumably reflects an evolutionary adaptation to a fluctuating dietary regimen.

The amino acid substitution mutation T756I in BT0867 enriched in the AD-evolved *B. theta* when under WD is particularly interesting. BT0867 is a starch utilization *(sus)C*-like gene in the polysaccharide utilization locus (PUL)12 that is important for mucin O-glycans digestion. *SusC* encodes the outer membrane proteins of *B. theta* that bind to and transport polysaccharides to facilitate digestion by the outer membrane and periplasmic enzymes.^45^ When AD clones containing such a mutation were cultured in the medium supplemented with mucin O-glycans, they not only outcompeted the ancestral strain but also had a higher growth rate than AD clones under SD. This finding demonstrates that WD selects mutations that promote the digestion of mucin-derived glycans to increase the gut colonization fitness of *B. theta* under a diet containing low MACs.

Of note, *B. theta* strains exhibit divergent *susC*/*susD* homolog gene pairs in their PUL for fructan utilization. The *susC/susD* pair in the PUL of *B. theta strain Bt-8736* enables the utilization of inulin, the homopolymer of β2-1 fructose units, whereas the pair in the PUL of *B. theta VPI-5482*, *Bt-3731*, and *Bt-7330* facilitates the utilization of levan, the homopolymer of β2-6 fructose units.^46^ As a result, *B. theta Bt-8736* outcompetes *B. theta VPI-5482* in the gut when dietary inulin is present.^46^ This finding suggests that, even in the presence of MACs, the type of MACs in the diet can differentially shape the gut microbiome function, and the *susC/susD* gene family is a key target of this selection. In line with this hypothesis, a longitudinal study tracing the within-person evolution of *B. fragilis* reveals that among a handful of genes under parallel adaptive evolution, which is a hallmark of positive selection, over one-third of them encode for homologs of SusC or SusD.^32^

### Mutation selection in *E. coli*

Studies using *E. coli* reveal that an increase in gut microbe colonization fitness can also result from loss-of-function mutations in the repressor of mucin utilization. Crook et al show that under a low-fiber diet, a probiotic *E. coli* Nissle strain, when mono-colonized in mice, accumulates loss-of-function mutations, such as premature stop codon, frameshift, and elimination of the start codon, which promote a metabolic shift toward mucin consumption.^47^ This functional shift is linked to mutations in the *nagC* gene, a repressor of the utilization of the mucin component N-acetylglucosamine (GlcNAc). Inactivation of *nagC* enables *E. coli* Nissle to consume GlcNAc under glucose-limiting conditions. Additional loss-of-function mutations were identified in *gntT*, which encodes a high-affinity gluconate transporter.^48^ Inactivation of *gntT* impairs gluconate utilization but enhances mucin metabolism, indicating that gluconate metabolism has an inhibitory effect on mucin utilization.^48^

In another study by Tsukimi et al, an *E. coli* K-12 hyper-mutator strain (Δ*mutL* or Δ*mutS*) was used to track mutations that facilitate *E. coli* colonization in germ-free mice under different diets.^41^ In the hyper-mutator *E. coli* strain, the *mut* genes that encode mismatch repair proteins that normally prevent genetic aberrations in the genome are mutated, which allows this strain to have more than 100-fold higher mutation frequencies than the wild-type strain.^49^ By tracking the mutations during *E. coli* colonization in mice fed high-MAC diets, they found notable loss-of-function mutations in sugar metabolism-related genes such as *araC*, *malI*, and *melR*, which encode a transcriptional factor that activates the arabinose operon, a repressor of the *malXY* operon, and a melibiose operon regulatory protein, respectively. These mutations enable *E. coli* to exhibit a higher competition index when exposed to a high-MAC diet compared with a low-MAC diet. This finding suggests that loss of function in these genes enhances *E. coli*'s ability to metabolize MACs, thereby improving its colonization fitness. Furthermore, the study shows that low-MAC and high-MAC diets have distinct impacts on the mutational trajectory of *E. coli*. In mice fed a high-MAC diet, mutations were predominantly selected in *mall*, *melR*, *ompC* (an outer membrane porin), and *ygjI* (an inner membrane transporter). In contrast, mice on a low-MAC diet selected mutations in *ssuC*, *nupG*, *cueO*, *elaD*, *gntR*, *yidE*, and *envZ*. The physiological importance of these mutations in colonization fitness, however, remains to be studied.

### Gene acquisition by horizontal gene transfer

The availability of MACs in the diet not only selects adaptive mutations but also promotes HGT events that exchange genes encoding carbohydrate-active enzymes. Groussin et al investigated HGT in the gut microbiota of individuals from industrialized and non-industrialized populations, finding that HGT occurs frequently within individuals.^50^ Although the transferred genes span a range of functions, individuals from non-industrialized populations, who typically consume more nondigestible fiber, exhibited a higher frequency of HGT involving carbohydrate-active enzyme-encoding genes. These observations support the notion that long-term consumption of a high-MAC diet enhances the carbohydrate-metabolizing capacity of the gut microbiota. This conclusion is consistent with the findings of Sonnenburg et al, who show that long-term low-MAC diets impair the gut microbiota's capacity to metabolize carbohydrates in a humanized mouse model.^34^

Collectively, these findings indicate that the availability of dietary MACs exerts a strong selective force on gut microbiome evolution. Acquiring genetic mutations or genes that enable efficient utilization of available nutrients, particularly carbohydrates, is critical for increasing the colonization fitness of gut microbes and promoting their functional diversification (Fig. 1).

**Figure 1:**
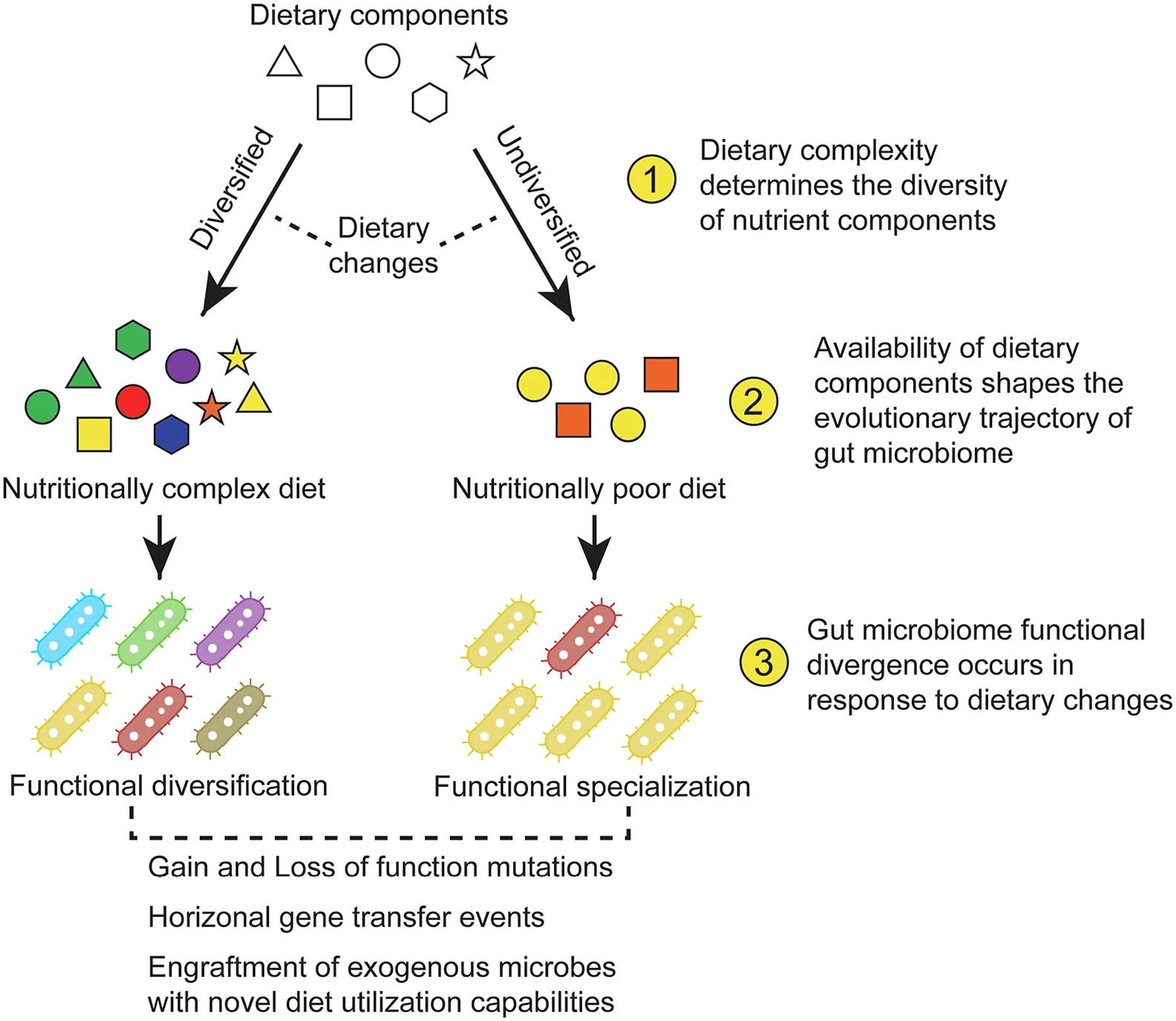
Model of how dietary complexity shapes the evolutionary trajectory of the gut microbiome. A nutritionally complex diet promotes the emergence of a functionally diversified gut microbiome, whereas a nutritionally poor diet leads to a functionally simplified gut microbiome. Multifaceted adaptive mechanisms are involved in shaping the metabolic capacities of the gut microbiome.

### The health impact

Gut microbes contribute to host health by fermenting dietary fibers and producing bioactive metabolites, among which short-chain fatty acids are the most extensively studied.^51^ Animal studies indicate that short-chain fatty acids enhance goblet cell differentiation and mucus-related gene expression and thereby enhance the mucus layer formation.^52^^,^^53^ The impenetrable colonic mucus layer is part of the mucosal barrier, and its integrity is critical for preventing gut inflammatory disorders such as inflammatory bowel diseases.^52^^,^^54^ A fiber-deficient diet reduces the production of short-chain fatty acids and shifts gut microbiota function toward host mucin-derived glycan degradation.^22^^,^^55^ Consequently, these changes compromise the colonic mucosal barrier,^55^, ^56^, ^57^, ^58^, ^59^ increasing the host's susceptibility to pathogen infection, inflammatory disorders, and colorectal cancer, as demonstrated in mouse studies. This may result in an increased risk of inflammatory disorders and colorectal cancer in humans. Moreover, emerging evidence indicates that low fiber diets are associated with an increased risk of other non-communicable diseases such as type 2 diabetes and cardiovascular diseases.^60^, ^61^, ^62^ The causal relationship between fiber-deficient diets, gut microbiota evolution, and disease risk remains to be further studied.

### The impact of high-fat and high-taurine intakes

In addition to a low dietary fiber intake, the Western-style diet contains a high amount of fat and taurine,^63^ and is associated with many chronic diseases, such as obesity, type 2 diabetes, cardiovascular diseases, and cancers.^64^^,^^65^ While the detailed mechanism by which a high-fat and high-taurine diet contributes to disease pathogenesis remains largely unclear, growing evidence suggests that it induces adverse changes in gut microbiome function and bile acid (BA) metabolism. To help the host digest and absorb fat, the liver synthesizes primary BAs and conjugates them with amino acids to generate bile salts.^66^^,^^67^ In humans, glycine or taurine-conjugated BAs are the most prominent bile salts. Bile salts are secreted into the duodenum to help emulsify and solubilize fats. Gut microbes deconjugate bile salts and transform them into secondary BAs. A high-fat intake increases the amount of BAs released into the intestine, thereby elevating the level of secondary BAs.^68^, ^69^, ^70^, ^71^, ^72^, ^73^ The bactericidal property of BAs selects gut microbes that are tolerant to them.^74^ Similarly, increased taurine intake elevates taurine-conjugated bile salts in the gut, leading to the selection of gut microbes capable of taurine deconjugation.^75^, ^76^, ^77^, ^78^ To adapt to these changes, gut microbes may employ strategies such as altering membrane permeability and enhancing their BA deconjugation capability.

### Membrane permeability alteration

BA-selected adaptive mutations are well-characterized in the *E. coli* OmpB operon, a two-component signal transduction system that regulates osmolarity-dependent gene expression.^79^ *E. coli* adapts to the fluctuating osmolarity in the intestine by modulating the diffusion pores in its outer membrane. The permeability of these pores is controlled by the differential expression of two porin proteins, OmpF and OmpC. Under low osmotic conditions, OmpF is predominantly expressed, as diffusion through OmpF-formed pores occurs at a much faster rate than through those formed by OmpC.^79^ The ratio of OmpF to OmpC is controlled by the OmpB operon that contains two key regulatory genes, *ompR* and *envZ*. The OmpR protein acts as a transcriptional regulator, and the intracellular level of its phosphorylated form determines the OmpF/OmpC ratio.^80^^,^^81^ EnvZ is an inner membrane sensor with dual kinase and phosphatase activities.^79^ Upon sensing the change in osmolarity, EnvZ modulates the level of phosphorylated OmpR, thereby altering the membrane pore size and permeability. When *E. coli* colonizes the mouse intestine, the high osmotic condition generated by BA selects missense mutations in the *envZ* gene that switch its protein activity toward an OmpR kinase-dominant state.^82^, ^83^, ^84^, ^85^ This leads to an elevated level of phosphorylated OmpR, which subsequently lowers the OmpF/OmpC ratio.^82^ Consequently, the evolved *E. coli* exhibits reduced membrane permeability and thereby enhanced resistance to bile salts compared with its ancestral strain.^82^

### Bile acid deconjugation

BA deconjugation requires gut microbe-produced bile salt hydrolases (BSHs), which catalyze the hydrolysis of amino acid-conjugated bile salts to yield deconjugated BAs.^86^ The latter then serve as precursors for microbe-mediated secondary BA transformation. As such, the BSH activity of the gut microbiome is a crucial gatekeeper for shaping the composition of the BA pool. BSH-encoded genes are widely spread in the gut microbiome by HGT.^18^^,^^87^, ^88^, ^89^, ^90^, ^91^, ^92^, ^93^, ^94^

Despite the broad distribution of BSH-coding genes in gut microbes, the catalytic efficiencies and substrate specificities of BSHs vary among microbes, even within the same species or strain.^86^ For instance, both *Lactobacillus acidophilus* (*L. acidophilus*) NCK1909 and *Lactobacillus gasseri* (*L. gasseri*) NCK2253 contain two BSHs, BshA and BshB.^95^ While both BSHs of *L. acidophilus* prefer deconjugating glycine-conjugated bile salts, the activity of its BshA is about 10 times greater than that of BshB.^95^ On the contrary, BshA and BshB of *L. gasseri* display distinct substrate specificities: BshA preferentially deconjugates taurine-conjugated bile salts, whereas BshB favors glycine-conjugated bile salts.^95^ Such differences in the catalytic efficiency and substrate specificity of BSHs presumably have evolved in response to the differential levels of exposure to glycine and taurine-conjugated BAs. Diet is a major environmental factor that determines the taurine to glycine conjugation ratio of BAs. A diet high in animal meat and seafood contains a much higher amount of taurine compared with a plant-based diet.^63^ Further, energy drinks in a Western-style diet increase the daily taurine intake by 6–16 times.^96^ A high intake of taurine results in an increase in taurine-conjugated bile salts and free taurine,^75^^,^^76^ and may consequently select gut microbes capable of deconjugating taurine and metabolizing taurine.^72^^,^^75^^,^^76^^,^^97^, ^98^, ^99^

How BSHs benefit microbial fitness in the intestine is not fully understood. Some studies support the hypothesis that deconjugated BAs are less toxic than their conjugated counterparts, which could promote gut microbial fitness.^18^^,^^100^ Other studies, however, suggest that the BSH activities of gut microbes are not associated with the resistance to BA toxicity,^101^^,^^102^ and the ultimate effects of BA deconjugation depend on the specific types of BAs present and the level of BSH activity.^95^ Nevertheless, it is clear that deconjugation of BAs enables microbial biotransformation of BAs, thus shaping the BA profile of the host. The transformed BAs act as signaling molecules to influence critical host physiological processes, such as metabolism, immune responses, tissue homeostasis, neurological function, and circadian rhythms.^103^, ^104^, ^105^ In this context, BSH activity in the gut microbiome facilitates the crosstalk between the host and gut microbiota, fostering a symbiotic relationship. In addition, amino acids released from BA deconjugation can serve as sources of nitrogen, carbon, and sulfur for some gut microbes,^105^ which increase their colonization fitness. Interestingly, a few recent studies have shown that gut microbes can conjugate BAs with amino acids other than glycine and taurine.^17^^,^^106^, ^107^, ^108^, ^109^, ^110^ However, the impact of such action on microbial fitness has yet to be investigated.

Altogether, these findings indicate that a higher intake of fat and taurine has a tremendous impact on gut microbiome composition and function due to the selective pressure imposed by the increased BA level and altered BA types.

### Health impact

As discussed above, a higher fat and taurine intake alters gut microbiota composition and BA metabolism. This leads to at least two known adverse effects. The first is the production of excessive hydrophobic secondary BAs, notably deoxycholic acid and lithocholic acid, which are toxic and tumorigenic and associated with the risk of hepatobiliary and colorectal cancers.^111^, ^112^, ^113^ Both secondary BAs induce inflammatory responses in steatotic HepG2 cells.^70^ Deoxycholic acid drives colon inflammation and stimulates intestinal cancer stem cell proliferation in a mouse model of colorectal cancer.^17^^,^^114^ Further, it suppresses the anti-cancer effector function of CD8^+^ T cells.^115^ Deoxycholic acid and lithocholic acid also impair cell junction and promote inflammatory macrophage response and vasculogenic mimicry, contributing to intestinal carcinogenesis.^116^, ^117^, ^118^ The second adverse effect of high-fat and high-taurine intake is increased taurine-conjugated bile salts in the gut.^75^, ^76^, ^77^, ^78^ Compared with unconjugated or glycine-conjugated bile salts, taurine-conjugated bile salts have a higher capacity for stimulating 7 alpha-dehydroxylation, a microbe-mediated biotransformation process to produce deoxycholic acids.^112^^,^^119^ This leads to an increased production of deoxycholic acids.^112^^,^^119^ Taurine deconjugation also increases the availability of free taurine, which increases the production of hydrogen sulfide, a genotoxicant.^119^ Notably, high fat also increases gut microbe-mediated non-classic amino acid conjugation of BAs that promote Wnt signaling and increase intestinal stem cell proliferation, known risk factors for colorectal cancer.^17^ As such, a higher fat and taurine intake results in a high production of pro-inflammatory and pro-tumorigenic secondary BAs and genotoxicants, which increases the risk for inflammatory and metabolic diseases and cancers. In line with this idea, BSH abundance in the gut microbiome is associated with intestinal and liver diseases, obesity, type 2 diabetes, cardiovascular diseases, and neurological diseases.^120^^,^^121^

### The impact of including specific diet components

The diet has been shown to be a predominant factor that shapes gut microbiota functional guilds composed of gut microbes with similar metabolic characteristics.^33^ Long-term consumption of a specific diet component drives the assembly of microbial functional guilds possessing the genetic capacity to utilize this substrate. A notable instance is the selection of gut microbes that degrade seaweed-derived polysaccharides.

The human gut microbiota harbors abundant bacteria capable of digesting fiber polysaccharides from commonly consumed terrestrial plants. For populations residing near coasts who historically consume macroalgae seaweeds, their gut microbes have gained the capability to digest algal polysaccharides, such as agarose and porphyran. Hehemann et al show that the gut *Bacteroides plebeians* (*B. plebeians*) in individuals in Japan possess a PUL that encodes the porphyranase, agarase, and the associated proteins required for degrading porphyran and agarose in seaweeds.^42^ In contrast, this specific PUL is absent in individuals in North America.^42^ Interestingly, genes in this PUL exhibit high sequence similarity to those found in *Zoebellia galactanivorans*, a member of marine *Bacteroidetes* isolated from algae.^42^ Along with this PUL, a mobile DNA element required for HGT was identified in *B. plebeians*, suggesting that these genes in gut *B. plebeians* were likely acquired from this marine bacterium through HGT as a result of the historical consumption of unsterilized seaweed in Japan.^42^^,^^43^

Additional evidence for the role of HGT in conferring microbial capacity to degrade algal polysaccharides comes from Pudlo et al, who have demonstrated that porphyran-degrading genes are widely distributed across the *Bacteroides* genus and linked to mobile DNA elements that mediate HGT.^43^ They show that the ability to degrade porphyrin and agarose has extended to Gram-positive Firmicutes in the gut. By conducting metagenomic surveys of human fecal samples taken in North and South America, Africa, Europe, and Asia, they show that the prevalence of seaweed-degrading genes is substantially higher in Chinese and Japanese subjects. On the contrary, subjects from the United States display an increased prevalence in the PUL of lambda-carrageenan, a sulfated polysaccharide extracted from red algae and often used as a food additive and historically consumed by European populations. These findings strongly support the hypothesis that consumption of seaweed selects gut microbes capable of digesting algal polysaccharides, and HGT plays an important role in acquiring PULs that build this capacity.

Intriguingly, Shepherd et al show that administering porphyrin to mice creates a metabolic niche that allows an exogenous *Bacteroides* strain harboring the porphyran utilization gene cluster to overcome priority exclusion by an isogenic competitor and engraft in the mouse gut microbiota.^122^ This finding suggests a potential evolutionary mechanism in which exogenous porphyran-degrading microbes are directly acquired in response to seaweed consumption.

### The health impact of introducing new diet components

The findings on gut microbiota adaptation to seaweeds suggest that dietary components influence the assembly and selection of microbial functional guilds possessing the genetic capacity to utilize specific substrates. From an evolutionary perspective, dietary diversification may promote the development of a metabolically versatile gut microbiome capable of adapting to fluctuating nutrient and carbon source conditions. Such metabolic flexibility may facilitate the production of a broader spectrum of bioactive metabolites that support host health by reducing the risk of inflammatory, metabolic, and neoplastic diseases. Consistent with this concept, greater dietary diversity during infancy and early childhood has been associated with reduced susceptibility to allergic diseases.^123^^,^^124^ Furthermore, there is a well-documented link between reduced gut microbial diversity and metabolic and inflammatory disorders and cancers.^125^, ^126^, ^127^ Despite these associations, the specific dietary components that most effectively promote microbiome functional diversity and confer health benefits remain largely undefined. Nevertheless, current evidence converges on the view that diets rich in plant-based, fiber-enriched foods and low in processed foods are associated with reduced disease risk (Fig. 2).

**Figure 2:**
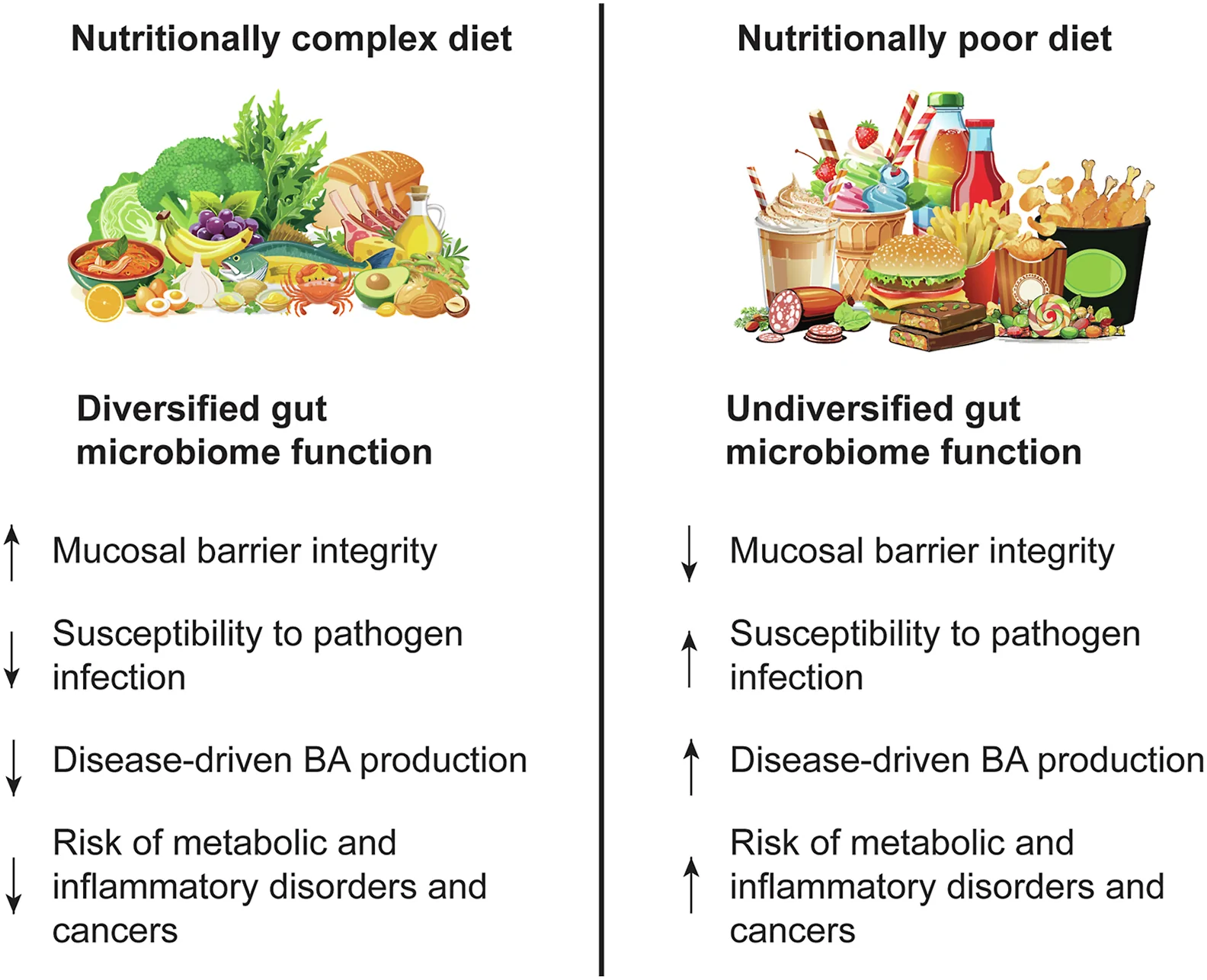
The health impact of nutritionally complex diets and poor diets. A nutritionally poor diet leads to a less diversified gut microbiome function and consequently increases the risk for many diseases because of reduced gut mucosal barrier integrity, increased susceptibility to pathogen infection, and elevated production of disease-driven bile acids (BAs).

## Conclusions and perspectives

Current evidence clearly demonstrates that host diet plays a crucial role in shaping gut microbiome evolution and function. Genetic adaptation of the gut microbiome in response to the host diet often enhances nutrient utilization and stress tolerance, ultimately improving colonization fitness. This can be achieved by changes at both the gut microbiota structural and gene levels. Multiple adaptive mechanisms, such as mutation selection, HGT, and acquisition of exogenous bacterial strains, have been found to contribute to expanding the metabolic capabilities of the gut microbiome (model illustrated in Fig. 1).

The modern lifestyle, such as consuming a high-fat, high-taurine, and low-fiber Western-style diet, has significantly altered microbiome functionality, increasing the susceptibility to metabolic and inflammatory disorders and cancers (Fig. 2). Despite these advances, our understanding of how diet influences the evolutionary trajectory of the gut microbiome and its implications for human health is still in its infancy. Many findings reviewed here rely on mono-associated mouse models or *in vitro* systems, which do not fully capture the complexity of gut microbial community effects. Moreover, the interplay between the diet and other host-specific factors such as sex, age, and genetics remains to be determined. Advancing such knowledge will aid in the rational design of diet-based personalized therapeutic strategies that prevent and treat a wide range of human diseases.

## CRediT authorship contribution statement

**Ka Lam Nguyen:** Writing – original draft, Investigation, Conceptualization. **Jason Sun Xia:** Validation. **Wenyan Mei:** Writing – review & editing, Validation, Supervision, Investigation, Funding acquisition, Conceptualization.

## Funding

This work was supported by the National Institute of Health (USA) (No. R01GM140306, R01ES036194 to W.M.).

## Conflict of interests

The authors declared no competing interests.
